# Technology-Based Interventions for Prevention of Type 2 Diabetes Following Gestational Diabetes: Systematic Review and Meta-Analysis

**DOI:** 10.2196/78841

**Published:** 2026-04-01

**Authors:** Claire Eades, Anh Nguyen-Hoang, Louise Hoyle, Dawn Cameron, Josie MM Evans

**Affiliations:** 1 Health Sciences Faculty of Health Sciences and Sport University of Stirling Stirling, Stirlingshire United Kingdom; 2 School of Health Sciences and Life Sciences University of the West of Scotland Lanarkshire, South Lanarkshire United Kingdom; 3 Public Health Scotland Edinburgh, Lothian United Kingdom

**Keywords:** diabetes prevention, gestational diabetes, health behavior change, mHealth, type 2 diabetes

## Abstract

**Background:**

Previous gestational diabetes incurs an 8-fold risk of developing type 2 diabetes, but lifestyle change can prevent or delay progression. Technology-based interventions may help overcome challenges women face in making postpartum lifestyle changes.

**Objective:**

This study aimed to assess whether technology-based diabetes prevention interventions improve outcomes related to the onset of type 2 diabetes among women with a previous diagnosis of gestational diabetes.

**Methods:**

Cochrane Central Register of Controlled Trials, CINAHL, Embase, PsycINFO, and Midwives Information and Resource Service were searched to October 2025 using subject headings and free-text terms. Titles and abstracts were independently screened by 2 authors, as were retrieved full-text articles. Studies were eligible if they examined technology-based diabetes prevention interventions delivered between gestational diabetes diagnosis and any time post partum, assessing anthropometric outcomes, glycemic control, health behavior, or psychological outcomes. Risk of bias was assessed by 1 reviewer using the National Institute for Clinical Excellence checklist, and certainty of evidence was assessed by 2 reviewers using the Grading of Recommendations Assessment, Development, and Evaluation. Data were summarized narratively, and results were pooled, where possible, using a random effects model.

**Results:**

This review identified 15 studies, including 1257 participants. Pooled analysis of 7 studies showed significantly greater weight loss among those receiving technology-based interventions (mean difference –1.01, SE 0.35, 95% CI –1.86 to –0.16 kg; *P*=.03). Interventions delivered using technology only showed increased weight loss (mean difference –1.13, 95% CI –3.12 to 0.86 kg) as did those with a longer follow-up (mean difference –1.58, 95% CI –3.93 to 0.76 kg) compared with combined technology and telemedicine approaches (mean difference –0.89, 95% CI –2.51 to 0.73 kg) and studies with shorter follow-up (mean difference –0.7, 95% CI –1.21 to –0.18 kg), but these differences were not significant (mode of delivery: *χ*^2^_1_=0.08; *P*=.78; follow-up: *χ*^2^_1_=1.06; *P*=.30). Meta-analysis showed no significant differences in BMI (mean difference –0.22, SE 0.1, 95% CI –0.4 to –0.01 kg/m^2^; *P*=.27; n=2 studies), fasting glucose (mean difference –0.03, SE 0.16, 95% CI –0.49 to 0.49 mmol/L; *P*=.99; n=4 studies), 2-hour glucose (mean difference 0.12, SE 0.19, 95% CI –0.47 to 0.72 mmol/L; *P*=.56; n=4 studies), hemoglobin A_1c_(mean difference –0.01%, SE 0.02%, 95% CI –0.24% to 0.23%; *P*=.74; n=2 studies), or homeostasis model assessment of insulin resistance (mean difference 0.07, SE 0.02, 95% CI –0.16 to 0.31; *P*=.16; n=2). Certainty of evidence for all pooled outcomes was very low.

**Conclusions:**

Technology-based interventions may help support women in reducing their risk of type 2 diabetes following gestational diabetes mellitus, but substantial heterogeneity, significant risk of bias, and very low certainty in the evidence mean that the findings should be interpreted cautiously. Trials with larger samples and longer follow-up are required to draw firm conclusions.

**Trial Registration:**

PROSPERO CRD42024324019; https://www.crd.york.ac.uk/PROSPERO/view/CRD42024324019

## Introduction

Diabetes is a growing global public health concern and a priority for preventive efforts [1]. The number of adults globally with diabetes in 2017 was estimated to be 451 million and is expected to rise to 693 million by 2045, with type 2 diabetes accounting for over 90% of cases [2]. Women who have had gestational diabetes mellitus (GDM) are at an 8-fold increased risk of developing type 2 diabetes [3]. GDM affects around 5% of pregnant women in Europe and is defined as glucose intolerance that is first diagnosed in pregnancy [4]. Although normal glucose regulation usually returns shortly after delivery, rates of type 2 diabetes after a diagnosis of GDM have been reported to be as high as 70% [5], with the greatest risk thought to be in the first 5 years following a pregnancy with GDM [6]. However, women who make positive lifestyle changes can prevent or delay progression to type 2 diabetes [7].

Challenges that women face in making lifestyle changes in the postpartum period include competing demands from work and home life, and a tendency to underestimate their personal risk and the severity of type 2 diabetes [8-10]. Technology-based interventions offer a possible solution to help to overcome some of these challenges by offering behavior change education and support remotely via messages, smartphone apps, or websites. A recent systematic review of technology-driven diabetes prevention interventions found that around two-thirds of included interventions were effective in achieving shorter-term weight loss and one-third were effective in achieving longer-term weight loss, but the review excluded studies focusing on participants with a previous diagnosis of GDM [11].

Although technology-driven interventions have been shown to be effective in supporting the management of GDM [12], to date, no reviews have been identified that focus on interventions primarily delivered using technology for diabetes prevention in women who have previously had GDM. A systematic review and meta-analysis carried out by Halligan et al [13] investigated the effectiveness of interventions with any technology or telemedicine component for women with previous GDM and found clinically relevant but non–statistically significant improvements in weight and BMI, but currently the effectiveness of primarily technology-based interventions is not known. The review by Halligan et al [13] included interventions with any technology component, and so for many of the included articles, the technology-based intervention was an adjunct to a telemedicine or face-to-face intervention. Telemedicine interventions involve real-time communication with a health care provider via telephone or video call, and although they have some practical benefits over a face-to-face appointment, telemedicine still relies on both parties arranging a convenient time for synchronous contact with a health care provider and so cannot offer the flexible access to advice and help that digital interventions can offer. Interventions delivered primarily using technology may offer the most benefits in terms of flexibility for women in the postpartum period and have the potential to be more cost-effective than telemedicine.

The review by Halligan et al [13] did not assess the behavior change techniques (BCTs) present in the interventions reviewed. BCTs are the active ingredients of an intervention that are designed to promote behavior change [14], and a taxonomy has been developed providing a list of standardized labels and definitions of BCTs. Using the shared language and descriptions provided by the BCT taxonomy allows the content of interventions to be unpicked and described in a consistent and comparable way, which may help to provide insight into the effective components of technology-based interventions. The objective of this review is to assess whether interventions focusing on diabetes prevention (not self-management of GDM), that are primarily technology-based, are effective in improving outcomes related to the onset of type 2 diabetes among women with a previous diagnosis of GDM and to identify and describe the BCTs present in interventions.

## Methods

### Overview

The systematic review and meta-analysis were conducted and are reported according to PRISMA (Preferred Reporting Items for Systematic Reviews and Meta-Analyses; Multimedia Appendix 1) guidelines [15], PRISMA abstract checklist (Multimedia Appendix 2) and the complementary PRISMA-S (Preferred Reporting Items for Systematic Reviews and Meta-Analyses literature search extension; Multimedia Appendix 3) for reporting literature searches within systematic reviews [16]. The protocol for the systematic review was registered with the International Prospective Register of Systematic Reviews, and no amendments were made to the protocol [17].

### Eligibility Criteria

The inclusion and exclusion criteria used to assess eligibility in the review are presented in Textbox 1.

**Inclusion and exclusion criteria.**

**Inclusion criteria**

- Adults aged 18 years or older who have received a previous diagnosis of gestational diabetes.
- Studies that examine the use of a technology-based intervention (eg, text, smartphone app, email, video, and website) in the antenatal or postpartum period with the stated aim of preventing or reducing the risk of type 2 diabetes.
- Interventions using both technology-based and face-to-face delivery, where the technology-based delivery is the primary means of intervention delivery and the technology-based intervention could feasibly be delivered as a stand-alone intervention.
- The outcomes of interest are anthropometric (weight, BMI, percent body fat, and waist or hip circumference), glycemic control (hemoglobin A_1c_, fasting plasma glucose, insulin resistance, prediabetes incidence/prevalence, and type 2 diabetes incidence), behavioral outcomes (diet, physical activity, or sedentary behavior), and psychological outcomes (beliefs about type 2 diabetes, self-efficacy for health behavior change, and behavioral intentions).
- Experimental and nonexperimental study designs, including observational studies, randomized trials, and nonrandomized trials.
- Studies published in English.

**Exclusion criteria**

- Studies of women who have received a diagnosis of type 1 or type 2 diabetes.
- Studies describing interventions where the technology-based component was only used to supplement a face-to-face intervention or to deliver real-time support with a human coach.
- Studies focusing on the self-management of gestational diabetes.
- Studies reporting only feasibility and acceptability related outcomes.
- Studies using a qualitative design only.

### Information Sources and Search Strategy

The databases MEDLINE, Central Register of Controlled Trials, CINAHL, Embase, PsycINFO, and Midwives Information and Resource Service were searched separately via the EBSCO platform in March 2024 using a combination of database subject headings and free-text words (Multimedia Appendix 4). No dedicated peer review of the search strategy was conducted. The search was rerun in October 2025 to check for papers published since the initial search in March 2024. No restrictions were applied for language, type, or date of publication, and no published search filters were used. No online resources, print sources, or citing references of included papers were searched. The reference lists of included papers were checked to identify any other potentially relevant papers, but experts in the field were not contacted due to the time-consuming nature of this process. Duplicates were removed in RefWorks (ProQuest LLC).

### Selection Process

The titles and abstracts of all articles were screened by 1 author (CE), and independent screening was split between 3 other authors, with DC screening one-quarter, LH screening one-quarter, and AN-H screening the other half. The full texts of papers were retrieved for studies that were considered relevant, but also for those that contained insufficient information to allow judgment of relevance. These were checked against the inclusion criteria by CE and independently by AN-H, LH, and DC. Where there were disagreements between reviewers about the inclusion of a paper, a consensus was reached through discussion among all authors.

### Data Collection Process and Data Items

A data extraction form was developed for this review and piloted on 3 papers, and then refined. Information was extracted by 1 reviewer (CE) under the following headings: authors, study location, type of intervention, mode of delivery, theory or model base for intervention, aim of the study, study design, inclusion criteria, size of the population, baseline characteristics, type of comparator, tailoring/individual patient–based, duration of intervention and measurement, outcome measures, measurement tools, sample size intervention group, sample size control group, dropout, findings summary, baseline outcomes intervention, baseline outcomes control, follow-up outcomes intervention, and follow-up outcomes control. Data were extracted for all available follow-up time points and outcomes available within each study. We attempted to contact authors where data were missing or not reported in the correct format for meta-analysis. If the data were not available or we received no response, studies were excluded from the meta-analysis. A second author (AN-H) independently checked all outcome data, and any errors were discussed and checked against the published paper by both CE and AN-H. The BCT taxonomy (version 1) [14] was used by 1 reviewer (AN-H) to code the BCTs present in the descriptions of included interventions. A second reviewer (CE) independently coded a random selection of 20% of interventions, and any disagreements were resolved by discussion.

### Risk of Bias Assessment

Internal and external validity of included studies was assessed by 1 reviewer (CE) using the National Institute for Clinical Excellence [18] checklist for quantitative intervention studies of any design. This checklist contains 27 items addressing 4 specific domains (population, method of allocation to intervention, outcomes, and analyses) and gives an appraisal of internal and external validity using a “++,” “+,” or “–“ rating scale depending on the extent to which criteria were satisfied, with a “++” indicating lowest risk of bias and “–“ highest risk of bias.

### Data Analysis

All data were grouped by outcome for synthesis and summarized narratively. Meta-analysis was planned for any outcome of interest recorded at both baseline and follow-up by 2 or more studies. Consistent with the Cochrane Collaboration guidance, only studies with a randomized controlled trial design were included in the meta-analysis [19]. After extraction, the following outcomes were synthesized using meta-analysis: weight, BMI, fasting plasma glucose, 2-hour glucose, hemoglobin A_1c_ (HbA_1c_), and homeostasis model assessment of insulin resistance (HOMA-IR). Results were pooled in SPSS (version 28; IBM Corp) using a random effects model to analyze the between-group differences in each outcome at follow-up. Forest plots were used to display individual study results and the syntheses. When there were follow-up data available at multiple time points, data from the last follow-up time point were used in the analysis. Where there were multiple publications reporting on the same sample, the data were only included in the analyses once to avoid double-counting of participants. When SD values were not reported, these were calculated from 95% CIs when possible. All pooled outcomes were continuous and were summarized using unstandardized mean difference with Sidik-Jonkman estimator and Knapp-Hartung SE adjustment as recommended for meta-analyses of a small number of studies [20]. Two-sided *P* values and 95% CIs were calculated for each outcome. Heterogeneity was quantified using between-study variance τ^2^. The *I*^2^ statistic is reported but interpreted with caution [21]. Prediction intervals were not calculated as the number of studies in the analyses fell below the recommended minimum number [22]. Analysis of publication bias was not conducted due to the small number of studies eligible for inclusion. Subgroup analyses were planned for the mode of intervention delivery (entirely digital or mixed) and length of follow-up (less than 6 months versus 6 months or more).

### Certainty Assessment

Certainty of evidence was evaluated using the Grading of Recommendations Assessment, Development, and Evaluation (GRADE; [23]) criteria for the main outcomes (weight, fasting glucose, 2-hour glucose, HbA_1c_, or HOMA-IR). GRADE classifies evidence into 4 levels: high, moderate, low, and very low based on assessment of risk of bias, inconsistency, indirectness, imprecision, and publication bias. With evidence rated as high quality, we can have very high certainty that the true effect is close to the estimated effect, whereas in evidence rated as very low, we can have almost no certainty in the estimates reported. Two reviewers (CE and LH) independently rated studies included in the meta-analysis using GRADEpro, and any disagreements were resolved by discussion. The table summarizing the results of the assessment can be found in Multimedia Appendix 5.

## Results

### Study Selection

**Figure 1:**
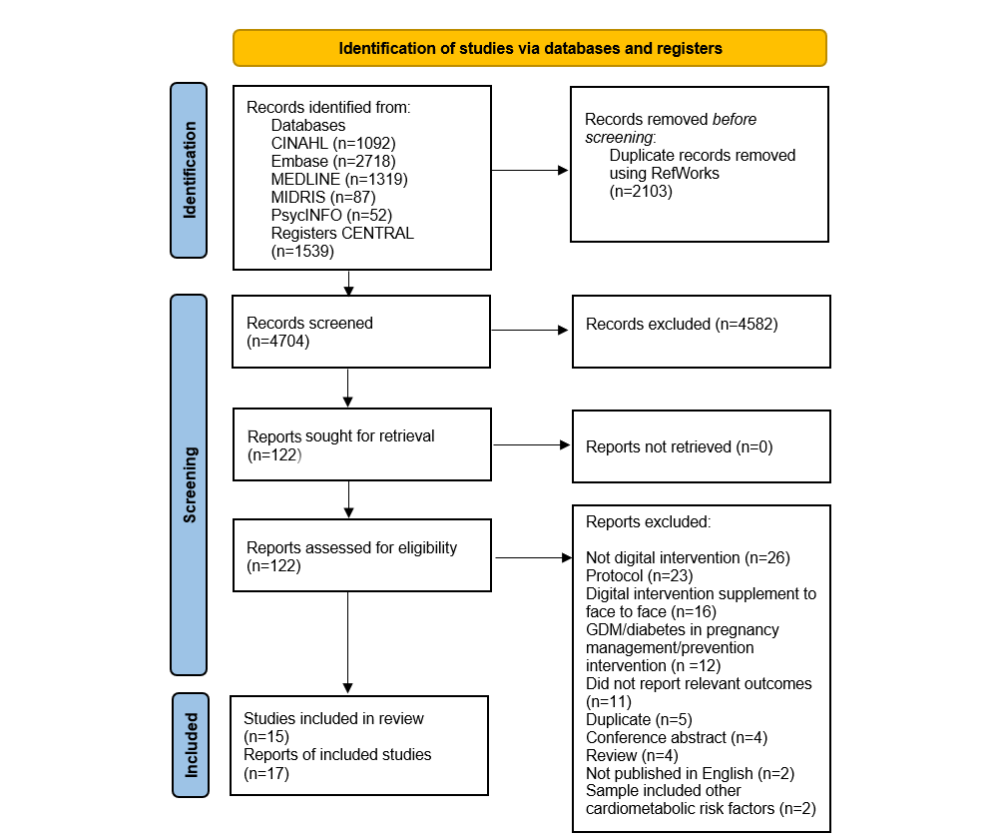
PRISMA (Preferred Reporting Items for Systematic Reviews and Meta-Analyses) flow diagram.

Figure 1 shows a PRISMA flow diagram of studies identified by the search. A total of 4704 papers were returned by the search, of which 4582 were excluded at the title and abstract screening stage. We reviewed the full text of 122 papers, and 15 studies [24-38] reported in 17 papers [39,40] were deemed eligible for inclusion in the systematic review. At the full-text screening stage, the following exclusions were made: 26 nondigital interventions, 23 protocols, 16 interventions where the technology-based component was a supplement to face-to-face, 12 interventions for the management or prevention of gestational diabetes, 11 papers that did not report relevant outcomes, 5 duplicates, 4 review papers, 2 papers not published in English, 2 papers that included women with additional cardiometabolic risk factors in the sample, and 1 conference abstract. A total of 4 conference abstracts were reviewed that did not contain enough information to assess eligibility. We searched the 4704 abstracts identified by our review by author name and intervention name, as outlined in the conference publications, to identify any further publications relating to these interventions. For 3 of the 4 conference abstracts, further publications with more information were identified that allowed assessment of eligibility. There were no related papers found for the fourth conference abstract, and no email addresses could be found for the authors through web searches, so the study was excluded from the review [41].

### Study Characteristics

The characteristics of the 15 included studies are presented in Table 1. Studies were published between 2012 and 2024, with almost two-thirds published between 2020 and 2024 (n=10, 66%). They were conducted in the United States (n=6) [28,31,32,34,36,37], Australia (n=4) [24,25,35,38], Asia (n=4) [26,28-30], and Europe (n=1) [33]. The mean age of participants among studies that reported age (n=13) [24-26,29-38] ranged from 31.5 to 40.4 years, with most means falling between 31 and 34 years (n=9, 69.2%). Study design was primarily randomized controlled trial (n=11) [24,25,27,29,30,32-36,38], with 2 studies adopting a before-and-after single-arm design [31,37] and 2 a nonrandomized controlled trial design [26,28].

**Table 1:** Summary of the characteristics of included studies.

Author (year), location	Enrolment setting	Design	Definition GDM^a^ used	IG^b^/CG^c^ sample size, n	Attrition IG/CG, %	Outcomes assessed	Follow-up duration	Risk of Bias	Age in IG (years), mean (SD)	Age in CG (years), mean (SD)	BMI in IG (years), mean (SD)	BMI in CG (years), mean (SD)
Cheung (2019), Sydney, Australia [24]	1 tertiary care hospital	RCT^d^	FPG^e^ ≥ 5.5 mmol/L and/or a 2hPG^f^ ≥ 8 mmol/L on 75 g OGTT^g^	40/20	28/35	Weight, PA^h^, diet	8.5 months	++/+	34 (4)	34 (4)	NR^i^	NR
Cheung (2024), Sydney, Australia [25]	3 tertiary care hospitals	RCT	FPG ≥ 5.5 mmol/L and/or a 2hPG ≥ 8 mmol/L on 75 g OGTT (at 2 hospitals) or one of the following: FPG ≥ 7.0 mmol/L; 2hPG ≥ 11.1 mmol/L on 75 g OGTT; random plasma glucose ≥ 11.1 mmol/L (at 1 hospital)	88/89	7/12	Weight, PA, diet	6 months	++/+	32.2 (4.6)	32.2 (4.6)	Prepregnancy: 29.4 (6.9)	Prepregnancy: 28.4 (5.9)
Ghaderi (2019), Tehran, Iran [26]	1 treatment center	Quasi-experimental with control group	NR	45/45	2/4	Risk perceptions for T2D^j^	NR	++/+	33.66 (3.93)	32.55 (4.44)	Prepregnancy: 26.44 (1.63)	Prepregnancy: 27.09 (2.13)
Kim (2012), Michigan, United States [27]	University health system (large nonprofit managed care plan with several private practices)	RCT	NR	28/21	7/23	Weight, WC^k^, FPG, 2hPG, PA, self-efficacy for diet and PA, risk perceptions type 2 diabetes	3 months	++/++	NR	NR	29.8 (6.8)	29.8 (6.8)
Kim (2021), South Korea [28]	3 hospitals	Quasi-experimental with control group	NR	64/64	8/3	Weight, body fat, FPG, HbA_1c_^l^, diet, health-promoting lifestyle profile, T2D knowledge	3 months	+/+	Over 35 45.6%	Over 35 50%	NR	NR
Liew (2023), Singapore [29]	NR	RCT	NR	31/30	10/6	Weight, BMI, FPG, 2hPG	2.5 months	++/+	36.5 (3.7)	35.4 (3.7)	24.0 (4.4)	22.7 (4.0)
Lim (2021), Singapore [30]	1 hospital	RCT	FPG ≥ 5.1 mmol/L and/or 1hPG^m^ ≥ 10.0 and/or 2hPG ≥ 8.5 mmol/L on 75 g OGTT	101/99	5/6	Weight, WC, diet, FPG, 2hPG, health directed behavior	4 months	++/+	32.6 (4.5)	32.4 (4.2)	Median 26.9 (IQR 23.0–29.5)	Median 25.8 (IQR 22.8–28.6)
Nicholson (2016), United States [31]	2 university-based prenatal clinics and 1 community-based practice	Single-arm pre-post study	NR	16/none	30	Weight, HbA_1c_, self-efficacy for PA	NR	+/++	31.5 (4.7)	NR	Prepregnancy: 29.4 (11.2)	NR
Nicklas (2014 [25], 2019 [39], 2020 [40]), Boston, United States	1 hospital	RCT	Meets ≥ 2 following 100 g OGTT: FPG ≥ 5.3 mmol/L, 1hPG ≥ 10.0 mmol/L, 2hPG ≥ 8.6 mmol/L, 3hPG^n^ ≥ 7.8 mmol/L. Or medical record documented clinician diagnosis	36/39	17/13	Weight, BMI, WC, FPG, 2hPG, HbA_1c_, HOMA-IR^o^, IGT^p^ and T2D prevalence, diet, PA	12 months	++/+	33.6 (4.8)	33.3 (5.8)	31.2 (5.8)	31.6 (5.5)
Potzel (2022), Germany [33]	University medical centre in Munich, Germany; Diabetes center in Dusseldorf; Institute for diabetes research in Tubingen	RCT	FPG ≥ 5.1 mmol/L and/or a 2hPG ≥ 8.5 mmol/L on 75 g OGTT	33/33	18/15	Weight, body fat, IGT prevalence, PA, diet, change in health habits	6 months	++/+	37.0 (4.4)	35.7 (3.1)	Median 26 (IQR 22.6–28.9)	Median 27.3 (IQR 23.9–30.3)
Reutrakul (2022), Chicago, United States [34]	NR	RCT	NR	9/6	0/17	Sleep duration, PA, FPG, 2hPG, area under the curve for glucose, HOMA-IR	1.5 months	+/++	42.0 (2.9)	38.7 (6.0)	32.7 (5.3)	32.6 (4.6)
Rollo (2020), New South Wales, Australia [35]	NR	3-arm RCT	NR	28 (15 low personalization [HP] and 13 low personalization [LP])/14	29/21	Weight, BMI, WC, body fat, HbA_1c_, diet quality, PA	6 months	++/++	HP^q^: 34 (4.5); LP^r^: 32.8 (3.6)	33.6 (3.8)	HP: 33.9 (3.6), LP: 33.9 (3.6)	31.1 (4.8)
Saxon (2023), Boston and Colorado, United States [36]	NR	RCT	Meets ≥ 2 following 100 g OGTT: FPG ≥ 5.3 mmol/L, 1hPG ≥ 10.0 mmol/L, 2hPG ≥ 8.6 mmol/L, 3hPG ≥ 7.8 mmol/L	91/90	48/49	Diet and PA self-efficacy, readiness to change	24 months	-/-	32.5 (4.9)	32.2 (5.5)	31.4 (5.9)	31.3 (5.8)
Seely (2020), Boston, United States [37]	Single federally qualified community health center	Single-arm pre-post study	NR	21/none	14	Weight, self-efficacy for PA and healthy eating	1.5 months	-/++	Whole sample: 33 (6.9)	NR	NR	NR
Taylor (2022), New South Wales, Australia [38]	NR	3-arm RCT	NR	48 (HP 25, medium personalization [MP] 23)/28	16/4	Weight, diet quality, PA, self-efficacy weight management	3 months	+/++	HP: 36.0 (5.4); MP^s^: 34.6 (5.3)	37.3 (5.3)	30.7 (4.3)	30.0 (4.8)

^a^GDM: gestational diabetes mellitus.

^b^IG: intervention group.

^c^CG: control group.

^d^RCT: randomized controlled trial.

^e^FPG: fasting plasma glucose.

^f^2hPG: 2-hour plasma glucose.

^g^OGTT: oral glucose tolerance test.

^h^PA: physical activity.

^i^NR: not reported.

^j^T2D: type 2 diabetes.

^k^WC: waist circumference.

^l^HbA1c: hemoglobin A_1c_.

^m^1hPG: 1-hour plasma glucose.

^n^3hPG: 3-hour plasma glucose.

^o^HOMA-IR: homeostatic model assessment of insulin resistance.

^p^IGT: impaired glucose tolerance.

^q^HP: high personalization.

^r^LP: low personalization.

^s^MP: medium personalization.

### Intervention Characteristics

Table 2 summarizes the characteristics of the interventions reported in the 15 included studies. Among the included studies, 2 [36,38] were full trials of interventions piloted in other studies included in the review [32,35]. Most interventions targeted diet and physical activity (n=12) [24,25,27-33,35,37,38]. Among the studies that did not target diet and physical activity, 1 targeted risk perceptions for type 2 diabetes [26], 1 addressed sleep [34], and 1 targeted self-efficacy for diet and physical activity [36]. Of the 12 studies targeting diet and physical activity behaviors, 7 also targeted additional behaviors or factors in the intervention, including breastfeeding [25,32], risk perceptions [27,31], self-efficacy [27], parenting [25], infant health/first aid [25,28], stress [28,29], sleep [29,33], and emotions [33]. A total of 4 studies stated that they were informed by 1 or more theories [25,31,33,37], with social cognitive theory the most frequently used (n=3). Among trials with a control arm (n=11), control participants were provided with information about gestational diabetes or diabetes prevention in 5 studies [24,28,32,33,36], received routine care in 4 studies [25-27,30], received the same app/website/devices as the intervention group but without messaging or coaching 2 studies [29,38], were on a waitlist for the intervention in 1 study [35], and received weekly emails with general health education and weekly brief telephone contact from a coach in 1 study [34].

**Table 2:** Summary of intervention characteristics.

Author (year)	Intervention name; description	Intervention duration	Time period intervention started	Intervention target	Mode of delivery	Control	Theoretical basis	Any tailoring
Cheung (2019) [24]	Smart Mums with Smart Phones; Lifestyle advice, education and support delivered via text messages and counseling, including feedback from activity monitor	8.5 months	Birth of baby	Diet, PA^a^	Text messaging (3 per week), provision of activity monitor, 2 × 30 min diet counseling sessions (1 remote and 1 face-to-face)	Leaflet	NR^b^	Text messages customized according to activity monitor data, breastfeeding status and ethnicity
Cheung (2024) [25]	Smart Mums with Smart Phones 2; Healthy lifestyle promotion delivered via text messages including feedback from activity monitor	6 months	Birth of baby	Diet, PA, breastfeeding, infant health, parenting	Text messaging (4 per week), activity monitor	Routine care	TRA^c^, SCT^d^, HBM^e^	Text messages customized to breastfeeding status and activity monitor data
Ghaderi (2019) [26]	NR; Education on risk of type 2 diabetes	NR	NR	Risk perceptions	Smartphone app	Routine care	NR	NR
Kim (2012) [27]	NR; Structured, web-based pedometer program giving personalized goals and education regarding lifestyle modification	3 months	Within 3 years of GDM diagnosis	Diet, PA, risk perceptions, self-efficacy	Website, text messaging, internet forum	Routine care	NR	Customized step count goals
Kim (2021) [28]	NR; Virtual reality program where various exercises could be viewed and performed and diet recorded	3 months	Birth of baby	Diet, PA, stress, infant first aid	Smartphone, virtual reality application and headset	Written educational materials on GDM^f^	NR	Adequacy of diet and calories were presented based upon BMI
Liew (2023) [29]	NR; Self-monitoring intervention using health tracker. Data from tracker reviewed by health coaches and personalized recommendations delivered by coaches	1 month	Within 10 years of GDM diagnosis	Diet, PA, sleep, stress	Smartphone app, continuous glucose monitor, health tracking ring, 2 sessions of chatroom messaging with health coach	Same app & devices as IG^g^ but no messaging	NR	Customized recommendations for diet, PA, sleep and stress
Lim (2021) [30]	SPAROW; App offering short videos on lifestyle, diet and activity tracking, personalized goals and live chat function	4 months	Birth of baby	Diet, PA	Smartphone app, live chat with health care professionals (5 to 10 min a day for first week then 2 to 3 times a week)	Routine care	NR	Customized weight and activity goals
Nicholson (2016) [31]	GooDMomS; Web-based lessons on healthy lifestyle, weight tracking and feedback on weight	7.5 months	From diagnosis of GDM	Diet, PA, risk perceptions	Website, 4 live webinars, message board, text messages/emails	NR	SCT	Customized feedback about weight, PA and diet
Nicklas (2014) [32]; (2019) [39]; (2020) [40]	Balance after Baby; Lifestyle modification adapted from DPP^h^ delivered via website and coach. Goals, weight and physical activity recorded on website	12 months	6 weeks post partum	Diet, PA, breastfeeding	Website, email and weekly telephone coaching for 12 weeks then every second week for 12 weeks and monthly thereafter	Leaflet	NR	NR
Potzel (2022) [33]	TRIANGLE; Self-paced DPP-based intervention with individualized challenges, chat coaching and behavior change technique library	6 months	3–18 months following GDM diagnosis	Diet, PA, sleep, emotions	Smartphone app, chat-based coaching	Leaflet	Kok’s taxonomy of behavior change methods	Customized and interactive challenges
Retrakul (2022) [34]	Sleep-Extend; Sleep tracker and app, didactic content and telephone coaching aimed at improving sleep	1.5 months	NR	Sleep	Smartphone app, wearable sleep tracker, email, weekly telephone coaching (5 to 20 min each)	Weekly health education emails (eg, vaccination for adults, cancer screening) & weekly brief telephone contact from the coach	NR	During coaching feedback given on sleep data, progress, and barriers explored and goals set
Rollo (2020) [35]	Body Balance Beyond; Website-based content on health lifestyles, goal setting, feedback on diet and activity from coaches, and text messaging support	6 months	Within 2 years of GDM diagnosis	Diet, PA	Website, text messaging, 6 coaching video calls (20–30 min each) with dietician and exercise physiologist	Waitlist control	NR	Coaching sessions reviewed diet and PA, explored barriers to diet and PA, refined goals and personalized strategies for achieving goals
Saxon (2020) [36]	Balance after Baby; See Nicklas 2014 description	12 months	6 weeks post partum	Self-efficacy and readiness to change for PA and diet	Website and weekly telephone, email and text messaging coaching for 12 weeks then every second week for 12 weeks and monthly thereafter	Access to websites with information about diabetes prevention	NR	NR
Seely (2020) [37]	Hola Bebé, Adiós Diabetes; Audio visual modules on healthy eating and physical activity delivered via app	2 months	Within 5 years of GDM diagnosis	Diet, PA	Smartphone app	NR	SCT	NR
Taylor (2022) [38]	Body Balance Beyond; See Rollo 2020 description	3 months	Within 5 years of GDM diagnosis	Diet, PA	Website, text messaging, 5 coaching video calls (20–30 min each) with a dietician or exercise physiologist	Access to intervention website only	NR	Coaching sessions reviewed diet and PA, explored barriers to diet and PA, set goals and strategies for achieving goals

^a^PA: physical activity.

^b^NR: not reported.

^c^TRA: theory of reasoned action.

^d^SCT: social cognitive theory.

^e^HBM: health belief model.

^f^GDM: gestational diabetes mellitus.

^g^IG: intervention group.

^h^DPP: diabetes prevention program.

The duration of interventions ranged from 1 to 12 months. In most studies, the intervention was carried out in the time period between diagnosis of GDM and 18 months post partum (8/13, 60% of studies reporting when the intervention was delivered) [24,25,28,30-33,36]. A total of 4 studies offered the intervention to women between a maximum of 2 and 5 years following diagnosis of GDM [27,35,37,38], and 1 study offered the intervention up to 10 years following diagnosis [29]. The technology-based modes of delivery used included smartphone apps [26,28-30,33,34,37], SMS text messaging [24,25,27,31,35,36,38], emails [31,32,34,36], websites [27,31,32,35,36,38], online message boards [27,31], virtual reality headset [28], activity/sleep trackers [24,25,34], webinars [31], and video calls [35,38], with most studies used multiple technology-based methods of delivery (n=11). A total of 7 studies used only technology-based modes of intervention delivery [25-30] and 8 used technology-based delivery plus synchronous contact with a health coach/interventionist through live chats [33], telephone calls [32,34,36], video calls [35,38], a combination of face-to-face and remote methods [24], or via webinars [31].

### Risk of Bias and Certainty of Evidence

A summary of the quality assessments can be found in Table 1. A total of 9 studies achieved a high-quality rating for internal validity, indicating that they minimized bias across multiple criteria [24-27,29,30,32,33,35]. Additionally, 6 studies received a high-quality rating for external validity, indicating that they minimized risk of bias in the generalizability of the study population across multiple criteria [27,31,34,35,37,38]. The GRADE assessment indicated that the certainty of evidence was very low for all pooled outcomes (weight, fasting plasma glucose, 2-hour plasma glucose, HbA_1c_, and HOMA-IR; Table 3).

**Table 3:** Grading of Recommendations Assessment, Development, and Evaluation assessment.

Outcome and certainty assessment									Patients, n			Effect			Certainty		Importance
	Studies, n	Study design	Risk of bias	Inconsistency	Indirectness	Imprecision	Other considerations	Technology-based interventions		Nontechnology-based interventions	Relative (95% CI)		Absolute (95% CI)				
**Weight (kg; follow-up: range 10-52 weeks)**																	
	7	Randomized trials	Serious^a^^,^^b^	Not serious	Serious^c^	Serious^d^	None	181		159	NR^e^		MD^f^ 1.01 SD lower (1.86-0.16 lower)	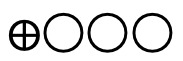 very low^a^^,^^b^^,^^c^^,^^d^		Critical	
**BMI (kg/m^2^; follow-up: range 10-13 weeks)**																	
	2	Randomized trials	Serious^a^^,^^b^	Not serious	Serious^c^^,g^	Serious^d^	None	47		51	NR		MD 0.22 SD lower (1.48-1.03 higher)	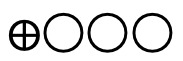 very low^a^^,^^b^^,^^c^^,^^d^^,^^g^		Critical	
**Fasting plasma glucose (follow-up: range 10-52 weeks)**																	
	4	Randomized trials	Serious^a^^,^^b^	Not serious	Serious^c^	Serious^d^	None	96		91	NR		MD 0 SD (0.5-0.49 higher)	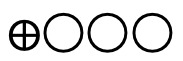 very low^a^^,^^b^^,^^c^^,^^d^		Critical	
**2-hour plasma glucose (follow-up: range 10-52 weeks)**																	
	4	Randomized trials	Serious^a^^,^^b^	Not serious	Serious^c^	Serious^d^	None	96		91	NR		MD 0.12 SD higher (0.47-0.72 higher)	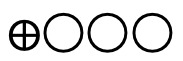 very low^a^^,^^b^^,^^c^^,^^d^		Critical	
**Hemoglobin A_1c_ (follow-up: range 26-52 weeks)**																	
	2	Randomized trials	Serious^a^^,^^b^	Not serious	Serious^c^	Serious^d^	None	54		53	NR		MD 0.01 SD lower (0.24-0.23 higher)	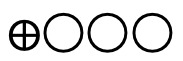 very low^a^^,^^b^^,^^c^^,^^d^		Critical	
**HOMA-IR^h^ (follow-up: range 6-52 weeks)**																	
	2	Randomized trials	Serious^a^^,^^b^	Not serious	Serious^c^	Serious^d^	None	48		44	NR		MD 0.07 SD higher (0.16-0.31 higher)	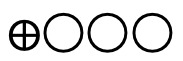 very low^a^^,^^b^^,^^c^^,^^d^		Critical	

^a^The nature of the technological interventions in this review (websites and smartphone apps) makes blinding difficult to achieve. A few studies managed to blind researchers and outcome assessors, but most studies did not blind participants or researchers or did not report in detail.

^b^Some studies did not adequately describe allocation concealment.

^c^Most studies are small, single region, pilot studies using surrogate/short-term outcomes which limits the directness of the evidence and applicability to the broader population of women with previous gestational diabetes.

^d^All studies have very small sample sizes of <400 and wide CIs.

^e^NR: not reported.

^f^MD: mean difference.

^e^Variation in interventions is present.

^h^HOMA-IR: homeostatic model assessment of insulin resistance.

### Anthropometric Outcomes

Weight was reported as an outcome in 12 studies [24,25,27-33,35,37,38], waist circumference in 4 [27,30,32,35], body composition in 3 [28,33,35], and BMI in 2 studies [27,29]. A total of 5 studies could not be included in the meta-analysis for weight: 2 were single-arm studies [31,37], 1 reported the proportion of women who met weight goals [25], 1 did not report SDs, and they could not be calculated [30], and 1 was a nonrandomized controlled trial [28]. Of the 5 studies that could not be pooled, only 1 [28] reported findings that favored the intervention in relation to weight loss.

**Figure 2:**
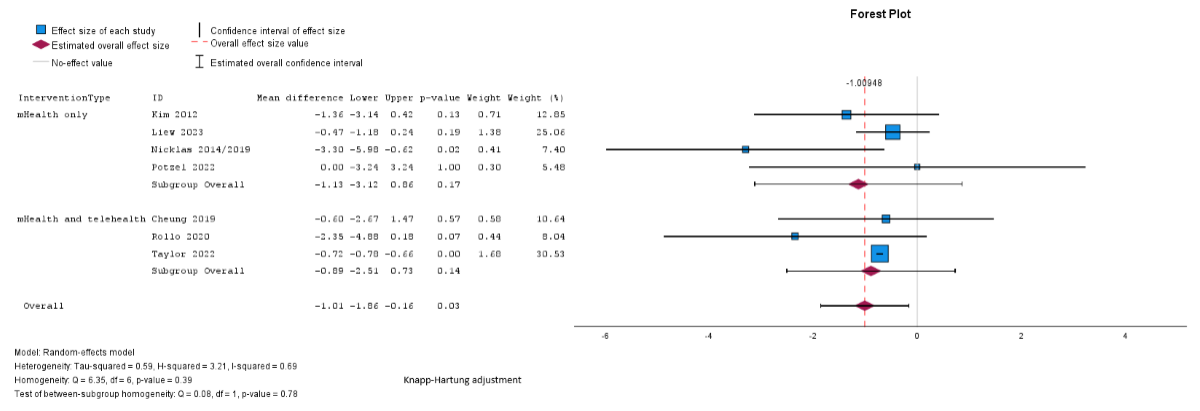
Random effects meta-analysis of the mean difference in weight (kg) comparing the intervention group and the control group according to the mode of delivery (digital only vs mixed) [24,27,29,32,33,35,38].

**Figure 3:**
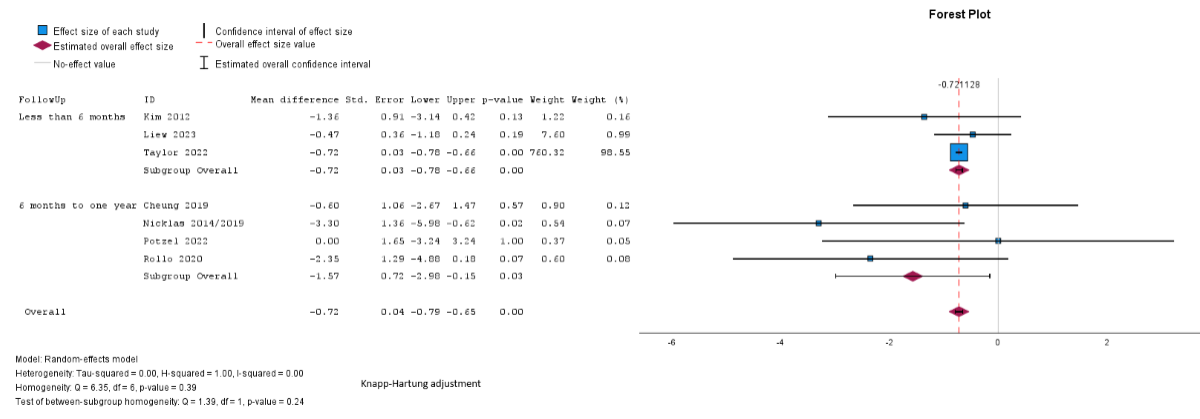
Random effects meta-analysis of the mean difference in weight (kg) comparing the intervention group and the control group according to the length of follow-up [24,27,29,32,33,35,38].

Pooled analysis showed significantly greater weight loss in the intervention group compared with the control (mean difference –1.01, SE 0.35, 95% CI –1.86 to –0.16 kg; *P*=.03; n=7; GRADE very low; Figure 2 [24,27,29,32,33,35,38]). τ^2^=0.59; τ=0.77; and *I*^2^=69% indicated higher heterogeneity. The effect did not remain significant across different modes of delivery and lengths of follow-up in subgroup analyses, although there was a larger reduction in weight noted among studies using technology-based delivery only (mean difference –1.13, 95% CI –3.12 to 0.86 kg for technology-based only, mean difference –0.89, 95% CI –2.51 to 0.73 kg for mixed delivery; Figure 2) and with a longer follow-up duration (mean difference –0.70, 95% CI –1.21 to –0.18 kg for follow-up <6 months; mean difference –1.58, 95% CI –3.93 to 0.76 kg for 6-12 months; Figure 3 [24,27,29,32,33,35,38]) but between-group differences were not significant for mode of delivery (*χ*^2^_1_=0.08; *P*=.78) or follow-up duration (*χ*^2^_1_=1.06; *P*=.30).

**Figure 4:**
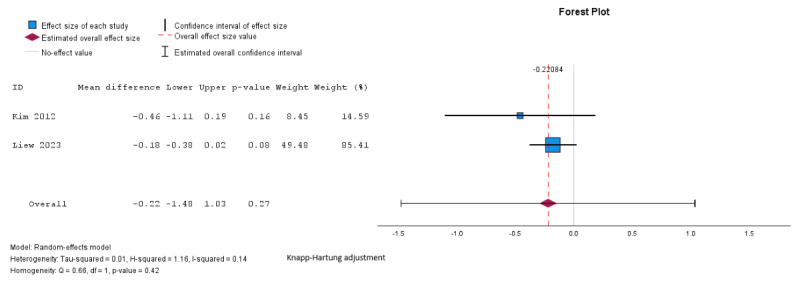
Random effects meta-analysis of the mean difference in BMI comparing the intervention group and the control group [27,29].

Pooled analysis showed a reduction in BMI in the intervention group compared to the control group, but the difference was not statistically significant (mean difference –0.22, SE 0.1, 95% CI –0.4 to –0.01 kg/m^2^; *P*=.27; n=2 [27,29]; GRADE very low; Figure 4 [27,29]). The *I*^2^=41%; τ=0.1; and τ^2^=0.01 indicated lower heterogeneity. Subgroup analyses were not possible for BMI because of the small number of studies assessing these outcomes and the uneven distribution of studies across the different subgroups.

None of the 4 studies assessing waist circumference reported significant differences between the intervention and control groups [27,30,32,35]. Of the 3 studies measuring body composition [28,33,35], only 1 found a significant reduction in body fat percentage in the intervention group compared to the control group [28]. There were too few studies reporting data on waist circumference and body composition at both baseline and follow-up to allow for meta-analysis of these outcomes.

### Glycemic Outcomes

Fasting glucose levels were reported in 5 studies [27-29,32,34], 2-hour glucose following an oral glucose tolerance test in 5 [27,29,30,34,39], HbA_1c_ in 4 [28,31,35,39], and HOMA-IR in 2 studies [34,39]. Only 1 study found a significant difference in glycemic outcomes, reporting lower fasting glucose and HbA_1c_ in the intervention group compared to the control group [28]. One study assessed the prevalence of impaired glucose tolerance [33] and another reported both impaired glucose tolerance and type 2 diabetes prevalence [32], with neither finding significant differences in prevalence among women receiving the intervention compared to the control group. Three studies reporting glycemic outcomes could not be pooled: SDs were not reported and could not be calculated for fasting and 2-hour glucose in 1 study [30], 1 study assessing fasting glucose and HbA_1c_ was a nonrandomized controlled trial [28], and another reporting HbA_1c_ was a single-arm design [31]. Subgroup analyses were not possible for glycemic outcomes because of the small number of studies assessing these outcomes and the uneven distribution of studies across the different subgroups of mode of delivery and length of follow-up.

**Figure 5:**
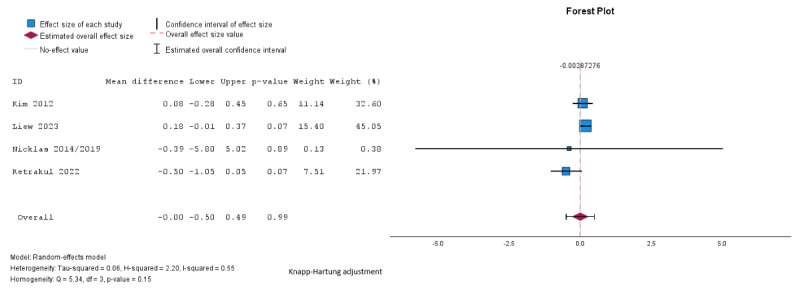
Random effects meta-analysis of the mean difference in fasting glucose comparing the intervention group and the control group [27,29,32,34].

**Figure 6:**
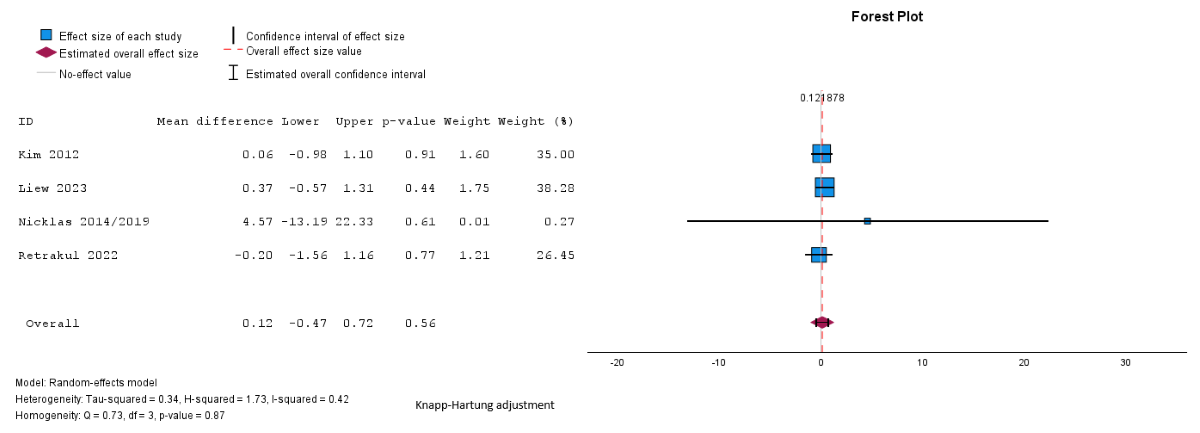
Random effects meta-analysis of the mean difference in 2-hour glucose comparing the intervention group and the control group [27,29,32,34].

**Figure 7:**
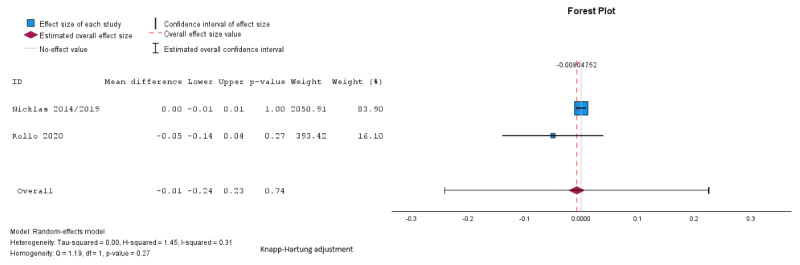
Random effects meta-analysis of the mean difference in hemoglobin A1c comparing the intervention group and the control group [32,35].

**Figure 8:**
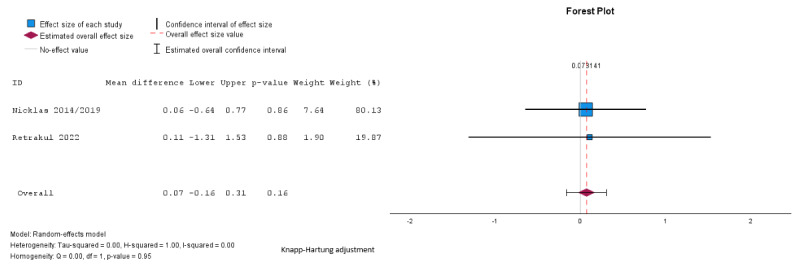
Random effects meta-analysis of the mean difference in homeostasis model assessment of insulin resistance comparing the intervention group and the control group [32,34].

Pooled analysis showed no significant difference between groups in fasting glucose (mean difference –0.03, SE 0.16, 95% CI –0.49 to 0.49 mmol/L; *P*=.99; n=4; GRADE very low; Figure 5 [27,29,32,34]), 2-hour glucose (mean difference 0.12, SE 0.19, 95% CI –0.47 to 0.72 mmol/L; *P*=.56; n=4; GRADE very low; Figure 6 [27,29,32,34]), HbA_1c_ (mean difference –0.01%, SE 0.02%, 95% CI –0.24% to 0.23%; *P*=.74; n=2 studies; GRADE very low; Figure 7 [32,35]), or HOMA-IR (mean difference 0.07, SE 0.02, 95% CI –0.16 to 0.31; *P*=.16; n=2; GRADE very low; Figure 8 [32,34]). The τ=0.24; τ^2^=0.06; and *I*^2^=55% indicate higher heterogeneity among the studies assessing fasting plasma glucose, and 2-hour glucose (*I*^2^=42%; τ=0.58; τ^2^=0.34), and lower heterogeneity in the studies assessing HbA_1c_ (*I*^2^=31%; τ=0.02; τ^2^=0.0004) and HOMA-IR (*I*^2^=0%; τ=0.001; τ^2^=0.000002).

### Behavioral Outcomes

A total of 8 studies reported diet outcomes [24,25,28,30,33,35,38,42], and 8 reported physical activity outcomes [24,25,27,32-35,38]. Only 1 study, testing an intervention aimed at improving sleep quality, reported any significant improvements in physical activity among women in the intervention group in comparison to the control group [34]. Two studies reported a significantly reduced calorie intake among those receiving the intervention compared to the control group [30,32], and 1 reported significantly more positive dietary habits among intervention participants [28]. Two studies reported on general measures of health-promoting lifestyles [28,30] and both found that the intervention group had significantly higher scores compared to the control group, indicating more healthy behaviors overall. Similarly, significantly more women in the intervention group of the study by Potzel et al [33] reported making changes to their health-related habits compared to the control group. It was not possible to synthesize behavioral outcomes using meta-analysis due to the variability in how outcomes were measured and reported.

### Psychological Outcomes

A total of 4 studies measured self-efficacy for physical activity [27,31,36,37], 3 self-efficacy for diet [27,36,37], and 1 study measured self-efficacy for weight management [38]. Two studies found significant differences in self-efficacy for physical activity [36,37], but in 1, the differences were accounted for by the fact that the control group’s self-efficacy worsened, whereas the intervention group remained stable [36]. One study found significant differences in self-efficacy for weight management [38], and 1 found significant differences in self-efficacy for diet [37]. Risk perceptions for type 2 diabetes were reported in 2 studies [26,27], with 1 finding significantly more changes in risk perceptions among women in the intervention group compared to the control group [26]. It was not possible to synthesize psychological outcomes using meta-analysis due to the variability in how these outcomes were measured and reported.

### BCTs

A total of 34 different BCTs were coded across the 15 included studies out of the 93 BCTs described in the BCT taxonomy. The number of BCTs in each study ranged from 6 to 17, with a mean of 11.3 (SD 2.82) per study. A 2-tailed independent samples *t* test found no significant difference in the mean number of BCTs in effective interventions (mean 10.5, SD 1.5) and noneffective interventions (mean 12.3, SD 3.7; *t*_7.7_=–1.19; *P*=.27). Table 4 summarizes the BCTs coded in all studies, and in effective vs noneffective interventions. The most commonly used BCTs were “instruction on how to perform the behavior,” which was included in all studies, “credible source” (14/15, 93% of studies), “self-monitoring of behavior” (13/15, 87%), “feedback on behavior” (11/15, 73%), “adding objects to the environment” (10/15, 66%), “prompts and cues” (10/15, 66%), “goal setting (behavior)” (10/15, 66%), “action planning” (10/15, 66%), “problem-solving” (9/15, 60%), and “information about health consequences” (8/15, 53%).

**Table 4:** Summary of behavior change techniques in effective and noneffective interventions.

Behavior change technique		All studies (n=15), n (%)	Effective any outcome (n=8), n (%)	Not effective for any outcome (n=7), n (%)
**Goals and planning**				
	Goal setting (behavior)	10 (66)	4 (50)	6 (86)
	Problem-solving	9 (60)	4 (50)	5 (71)
	Goal setting (outcome)	2 (13)	2 (25)	0 (0)
	Action planning	10 (66)	5 (63)	5 (71)
	Review behavior goal(s)	6 (40)	2 (25)	4 (57)
	Discrepancy between current behavior and goal	1 (7)	0 (0)	1 (14)
	Review outcome goals	1 (7)	1 (13)	0 (0)
**Feedback and monitoring**				
	Feedback on behavior	11 (73)	5 (63)	6 (86)
	Self-monitoring of behavior	13 (87)	7 (88)	6 (86)
	Self-monitoring of outcomes of behavior	4 (27)	3 (38)	1 (14)
	Feedback on outcomes of behavior	1 (7)	0 (0)	1 (14)
**Social support**				
	Social support (unspecified)	9 (60)	3 (38)	6 (89)
	Social support (practical)	2 (13)	2 (25)	0 (0)
	Social support (emotional)	1 (7)	1 (13)	0 (0)
**Shaping knowledge**				
	Instruction on how to perform the behavior	15 (100)	8 (100)	7 (100)
**Natural consequences**				
	Information about health consequences	8 (53)	4 (50)	4 (57)
	Salience of consequences	3 (20)	3 (38)	0 (0)
	Information about social and environmental consequences	4 (27)	2 (25)	2 (29)
	Information about emotional consequences	1 (7)	1 (13)	0 (0)
**Comparison on behavior**				
	Demonstration of the behavior	7 (47)	6 (75)	1 (14)
	Social comparison	1 (7)	0 (0)	1 (14)
**Associations**				
	Prompts/cues	10 (66)	6 (75)	4 (57)
**Repetition and substitution**				
	Behavioral practice/rehearsal	2 (13)	2 (25)	0 (0)
	Habit formation	1 (7)	1 (13)	0 (0)
	Graded tasks	2 (13)	0 (0)	2 (29)
**Comparison on outcomes**				
	Credible source	14 (93)	8 (100)	6 (86)
	Pros and cons	1 (7)	0 (0)	1 (14)
**Reward and threat**				
	Material reward (behavior)	1 (7)	0 (0)	1 (14)
	Nonspecific reward	1 (7)	0 (0)	1 (14)
	Social reward	5 (33)	2 (25)	3 (43)
**Regulation**				
	Reduce negative emotions	1 (7)	0 (0)	1 (14)
**Antecedents**				
	Restructuring the physical environment	1 (7)	0 (0)	1 (14)
	Adding objects to the environment	10 (66)	7 (88)	3 (43)
**Identity**				
	Framing/reframing	4 (27)	1 (13)	3 (43)

There were 6 BCTs identified as being present in 75% (6/8) or more of effective interventions: “self-monitoring of behavior,” “instruction on how to perform the behavior,” “demonstration of the behavior,” “prompts/cues, credible source,” and “adding objects to the environment.” However, these BCTs were all present in very similar proportions of the studies that were not effective, except for “demonstration of the behavior,” which was found only in 14% (1/7) of noneffective studies compared to 75% (6/8) of effective ones, and “adding objects to the environment,” which was present in 43% (3/7) of noneffective interventions compared to 88% (7/8) of effective interventions.

Differences between effective and noneffective interventions were noted for 3 other BCTs: “goal setting (behavior),” “social support (unspecified),” “salience of consequences.” The “goal setting (behavior)” BCT was used more frequently in noneffective interventions (6/7, 86%) than in effective interventions (4/8, 50%). Effective interventions less frequently used the BCT “social support (unspecified)” (3/8, 38%) compared to noneffective interventions (6/7, 86%), but more frequently used practical (2/8, 25%) or emotional social support (1/8, 13%), which were not used in any noneffective interventions. The “salience of consequences” BCT was used in 38% (3/8) of effective interventions and not used in any noneffective interventions. However, all of the comparisons between effective and noneffective interventions must be interpreted with caution due to the small number of studies in each group.

## Discussion

### Principal Findings

This systematic review identified 15 studies [24-38] reported in 17 papers [39,40], investigating interventions aiming to prevent type 2 diabetes among women with a history of gestational diabetes using primarily technology-based delivery. Weight loss was the most frequently assessed outcome [24,25,27-33,35,37,38], but other outcomes related to diabetes risk assessed included waist circumference [27,30,32,35], body composition [28,33,35], BMI [27,29], glycemic control [27-32,34,35,39], health behaviors [24,25,27,28,30,32-35,38], self-efficacy for behavior change [27,31,36-38], and risk perceptions of type 2 diabetes [26,27]. Only 1 study assessed type 2 diabetes prevalence and found no significant differences between the intervention and control group [32]. Pooled analysis of 7 studies [24,27,29,32,33,35,38] revealed a significantly greater weight loss among those receiving technology-based interventions. Interventions delivered using technology [27,29,32,33] and with a longer follow-up [25,32,33,35] resulted in increased weight loss compared to those using combined technology and telemedicine approaches [24,35,38] and studies with a shorter follow-up [27,29,38]. Meta-analysis showed no significant differences in BMI [27,29], fasting glucose [27,29,32,34], 2-hour glucose [27,29,32,34], HbA_1c_ [32,35], or HOMA-IR [32,34] between intervention and control groups. There was heterogeneity in the study populations, dropout rates, target, timing, and duration of the intervention, and modes of delivery. Comparable heterogeneity has been reported in other systematic reviews of technology-based or telemedicine interventions for diabetes prevention [11,13].

### Comparison With Other Literature

Although most studies included in this review did not individually report significant effects of technology-based interventions on weight loss, pooled analysis revealed a significantly greater weight loss among those receiving technology-based interventions with a mean difference of –1.01 (95% CI –1.86 to –0.16) kg. Although this difference is relatively small, 1 study found that weight loss of 1 kg in 1079 participants of a diabetes prevention intervention reduced the risk of type 2 diabetes by 16% over 3 years [43]. A systematic review of primarily face-to-face lifestyle interventions for women with a history of gestational diabetes reported a mean difference of –1.06 (95% CI −1.68 to −0.44) kg in pooled analysis of 5 trials, suggesting that technology-based interventions may be close to face-to-face interventions in effectiveness for weight loss in this population [44], but with possible benefits in terms of the resources required for delivery. Research has supported the cost-effectiveness of digital health interventions for supporting behavioral change in other populations [45,46], and so it is possible that technology-based interventions could offer a cost-effective means of supporting lifestyle change among women following a diagnosis of GDM. However, none of the studies in this review included any economic analysis, and this should therefore be explored in future trials.

Subgroup analyses carried out in this meta-analysis showed that interventions delivered using technology-based methods only resulted in more weight loss compared to those interventions using a combination of technology-based and telemedicine approaches, but these differences were not statistically significant. Studies with a follow-up of 6 months or more also resulted in increased weight loss than those with a shorter follow-up, which is consistent with a previous systematic review by Goveia et al [47] that included both technology-based and face-to-face lifestyle interventions for diabetes prevention in women with GDM. Intervention length and follow-up length in studies in this review were largely similar, and so these findings suggest that technology-based interventions for this population should have a duration of 6 months or more to maximize effectiveness. The need for help over a longer period is supported by women’s descriptions of the challenges of managing lifestyle change alongside the multiple roles they fulfill in the postpartum period [10].

The findings in relation to glycemic outcomes were less positive than those for weight. Only 1 of the 5 studies assessing glycemic control reported significant differences favoring the intervention group, and pooled analyses reported no significant differences in fasting glucose, 2-hour glucose, HbA_1c_, or HOMA-IR. The review by Goveia et al [47] also found no significant effect of lifestyle intervention on fasting and plasma glucose but did find a positive effect for HbA_1c_. Goveia et al [47] found a reduction in the incidence of type 2 diabetes, which was larger in interventions that started within 6 months of women giving birth. Only 1 study in this review assessed type 2 diabetes and found no significant differences in prevalence between the intervention and control group, but the intervention started up to 3 years postdelivery in this study.

It was not possible to pool studies on physical activity and diet in this review, but individually, most studies did not show significant effects of the intervention on either outcome. In contrast, half of the studies assessing self-efficacy for physical activity and diet, or risk perceptions, reported significant differences that favored the intervention group. This suggests that technology-based interventions included in this review do appear to be capable of changing the antecedents of behavior, even if studies do not demonstrate significant changes in lifestyle behavior. However, it is difficult to draw conclusions from individual studies in this review due to the limitations of the studies in relation to sample size, as discussed in the Limitations section below.

There was no significant difference in the number of BCTs in effective studies compared to noneffective studies in this review. This is in contrast to the findings of a systematic review of technology-driven type 2 diabetes prevention interventions in high-risk groups, excluding women with GDM, by Van Rhoon et al [11], which found that effective interventions had more BCTs on average. In our review, there was an average of 11.3 (SD 2.82) BCTs across all interventions compared to an average of 9 in the interventions included in Van Rhoon et al’s review [11]. It has been argued that it is important to include fewer, more effective BCTs in technology-based interventions where attrition rates can be high, and too many BCTs might make the intervention more time-consuming and less engaging [48]. Research on BCTs in technology-based interventions aiming to promote physical activity and reduce sedentary behavior in adults suggests that the specific combination of BCTs may also be important, with certain combinations being more effective than others [48]. Given that there was no difference in the number of BCTs between effective and noneffective interventions in this review, and the challenges and competing demands that women report when attempting lifestyle change in the postpartum period [10], future research should arguably focus on what BCTs are likely to be effective in women with GDM, rather than the quantity of BCTs.

There was little difference in the BCTs contained in effective interventions and noneffective interventions in this review except for “demonstration of behavior,” “adding objects to the environment,” and specific types of “social support” (eg, “emotional or practical”) being more common in effective interventions, whereas less effective interventions were more likely to contain “unspecified social support.” Furthermore, descriptions of interventions were often lacking in detail, and these differences in social support may represent differences in reporting rather than true differences in intervention approaches. Clearer specification of interventions is needed in trials to build a cumulative evidence base of effective interventions that can be replicated and refined.

The most commonly included BCTs across all studies in this review were: “instruction on how to perform the behavior,” “credible source,” “self-monitoring of behavior,” “feedback on behavior,” “adding objects to the environment,” “prompts and cues,” “goal setting (behavior),” “problem-solving,” “action planning,” and “information about health consequences.” The systematic review of technology-driven type 2 diabetes prevention interventions in high-risk groups, excluding women with GDM, by Van Rhoon et al [11] found that 90% of effective interventions included the BCTs “social support (unspecified),” “goal setting (outcome/behavior),” “feedback on behavior,” and “self-monitoring of outcomes” [11]. Of these, “social support (unspecified),” “goal setting (outcomes),” and “self-monitoring (outcomes)” were not among the most used BCTs in this review, and future research should consider the inclusion of these BCTs and assess their transferability from other high-risk groups to women with previous GDM.

### Limitations

Limitations of the review include that non-English papers were excluded, gray literature was not identified, and data were not independently extracted but instead checked by a second reviewer. Prediction intervals could not be calculated in the meta-analysis because of the small number of eligible studies. By reporting only CIs and not prediction intervals, we cannot draw conclusions about the possible variation in the intervention effect across different populations and settings [22]. Key limitations of the studies included in the review were the small sample sizes and lack of power calculations [49]. Sample sizes in all included studies were very small, with all studies containing around 100 participants or fewer in each arm. Two-thirds of studies included in the review either did not report carrying out a power calculation or reported being underpowered. Given that meta-analysis showed significant reductions in weight favoring the interventions when most individual studies did not, it is possible that interventions did result in behavioral changes, but studies were not adequately powered to detect these differences [49]. The certainty of the evidence for all pooled outcomes in this review was rated as very low.

Interventions were often poorly described, making it difficult to code and identify potentially effective ingredients, and most lacked an explicit theoretical basis to explain and understand how they might work. The explicit use of theory in interventions may increase the likelihood that an intervention will be effective by ensuring that the causal determinants of the behavior are understood and addressed in the intervention [50]. There were not enough studies using theory in this review to suggest a particular model or framework that should be used, but instead, we recommend that, to maximize the potential effectiveness of technology-based interventions in this group and facilitate replicability, future research should clearly describe the content of interventions and consider the theoretical basis for the intervention. There was also a variety in the mode of delivery of digital interventions, and future research should explore whether providing participants with additional devices (eg, fitness trackers) offers any significant benefit over them using their own devices (eg, smartphones) that outweighs the costs of these devices.

### Conclusions

This review is the first to summarize interventions with primarily technology-based delivery to prevent type 2 diabetes in women following a diagnosis of GDM. Previously published reviews included interventions with any digital component, and in many of the studies reviewed, the digital aspect was an adjunct to in-person or telemedicine delivery, meaning that conclusions could not be clearly drawn about the benefit of primarily digital interventions [13]. This review is also the first to code the BCTs in interventions with the aim of preventing type 2 diabetes in this population, and so can suggest techniques that should be explored in future interventions. The findings of the review suggest that there may be potential for technology-based interventions to support women in reducing their risk of type 2 diabetes following GDM. However, substantial heterogeneity, significant risk of bias, and very low certainty in the evidence according to the GRADE assessment mean that the findings must be interpreted cautiously. Further, high-quality trials with larger samples, longer follow-up, and clearer reporting of interventions are required to allow firm conclusions to be drawn.

## Appendix

Multimedia Appendix 1 PRISMA checklist.

Multimedia Appendix 2 PRISMA abstract.

Multimedia Appendix 3 PRISMA-S checklist.

Multimedia Appendix 4 Full search strategy.

Multimedia Appendix 5 Data file.

## Abbreviations

BCT: behavior change technique
GDM: gestational diabetes mellitus
GRADE: Grading of Recommendations Assessment, Development, and Evaluation
HbA1c: hemoglobin A1c
HOMA-IR: homeostasis model assessment of insulin resistance
PRISMA: Preferred Reporting Items for Systematic Reviews and Meta-Analyses
PRISMA-S: Preferred Reporting Items for Systematic Reviews and Meta-Analyses literature search extension

## Data Availability

The datasets generated during this study are available in the additional supporting files for this study.
