# Supplementary material for: Technology-Based Interventions for Prevention of Type 2 Diabetes Following Gestational Diabetes: Systematic Review and Meta-Analysis
Source: J Med Internet Res. 2026 Apr 1;28:e78841. doi: 10.2196/78841 (PMC13085990; doi:10.2196/78841)
Supplement: Multimedia Appendix 4 [file jmir_v28i1e78841_app4.docx]

MEDLINE

1. Diabetes, Gestational/
2. Gestational diabetes.ti
3. Pregnancy induced diabetes.ti
4. Gestational diabetes mellitus.ti
5. GDM.ti
6. Pregnancy diabetes mellitus.ti
7. 1/6 OR
8. Health education/
9. Patient Education as Topic/
10. Preventative Medicine/
11. Risk/
12. Risk reduction behaviour/
13. Education*.ti
14. Intervention*.ti
15. Prevent* .ti
16. Program* .ti
17. Risk adj reduc*.ti
18. Risk.ti
19. 8/18 OR
20. Cell Phone/
21. Computers/
22. Computers, Handheld/
23. Digital Technology/
24. Education, Distance/
25. Electronic Mail/
26. Fitness Trackers/
27. Internet/
28. Mobile Applications/
29. Multimedia/
30. Online Social Networking/
31. Smartphone/
32. Social Media/
33. Social Networking/
34. Software/
35. Telemedicine/
36. Telephone/
37. Television/
38. Text Messaging/
39. Video Games/
40. Video-Audio Media/
41. Virtual Reality/
42. Wearable Electronic Device/
43. Web browser/
44. Webcast/
45. App*.mp
46. Computer.mp
47. Digital.mp
48. DVD*.mp
49. eHealth.mp
50. e-Health.mp
51. Electronic.mp
52. eMail*.mp
53. Internet.mp
54. mHealth.mp
55. m-Health.mp
56. Mobile.mp
57. Multimedia.mp
58. Online.mp
59. Pedometer*.mp
60. Phone.mp
61. Sensor*.mp
62. Smartphone.mp
63. SMS.mp
64. Social media.mp
65. Social adj media.mp
66. Social adj network*.mp
67. Software.mp
68. Technolog*.mp
69. Telehealth.mp
70. Telemedicine.mp
71. Telephone.mp
72. Television.mp
73. Text.mp
74. Tracker*.mp
75. TV.mp
76. Video*.mp
77. Virtual.mp
78. (virtual or online) adj (communit* or network*).mp
79. Wearable*.mp
80. Web*.mp
81. 20/80 OR
82. 7 AND 19 AND 81

CENTRAL searc

1. Diabetes, Gestational
2. Gestational diabetes:ti
3. Pregnancy induced diabetes:ti
4. Gestational diabetes mellitus:ti
5. GDM:ti
6. Pregnancy diabetes mellitus:
7. 1/6 OR
8. Health education/
9. Patient Education as Topic/
10. Preventative Medicine/
11. Risk/
12. Risk reduction behaviour/ (
13. Education*:ti
14. Intervention*:ti
15. Prevent*:ti
16. Program*:ti
17. Risk NEAR reduc*:ti
18. Risk:ti
19. 8/18 OR
20. Cell Phone/
21. Computers/
22. Computers, Handheld/
23. Digital Technology/
24. Education, Distance/
25. Electronic Mail/
26. Fitness Trackers/
27. Internet/
28. Mobile Applications/
29. Multimedia/
30. Online Social Networking/
31. Smartphone/
32. Social Media/
33. Social Networking/
34. Software/
35. Telemedicine/
36. Telephone/
37. Television/
38. Text Messaging/
39. Video Games/
40. Video-Audio Media/
41. Virtual Reality/
42. Wearable Electronic Device
43. Web browser/
44. Webcast/
45. App*
46. Computer
47. Digital
48. DVD*
49. eHealth
50. e-Health
51. Electronic
52. eMail*
53. Internet
54. mHealth
55. m-Health
56. Mobile
57. Multimedia
58. Online
59. Pedometer*
60. Phone
61. Sensor*
62. Smartphone
63. SMS
64. Social media
65. Social NEAR media
66. Social NEAR network*
67. Software
68. Technolog*
69. Telehealth
70. Telemedicine
71. Telephone
72. Television
73. Text
74. Tracker*
75. TV
76. Video*
77. Virtual
78. (virtual or online) NEAR (communit* or network*)
79. Wearable*
80. Web*
81. 20/80 OR
82. 7 AND 19 AND 81

CINAHL search

1. Diabetes Mellitus, Gestational/
2. “Gestational diabetes”.ti
3. “Pregnancy induced diabetes”.ti
4. “Gestational diabetes mellitus”.ti
5. GDM.ti
6. “Pregnancy diabetes mellitus”.ti
7. 1/6 OR
8. Diabetes Education/
9. Health Education/
10. Patient Education/
11. Education*.ti
12. Intervention*.ti
13. Prevent*.ti
14. Program*.ti
15. Risk N reduc*.ti
16. Risk.ti
17. 8/16 OR
18. Cellular Phone/
19. Computers and Computerization/
20. Computers, Hand-held/
21. Computers, Portable/
22. Email/
23. Fitness Trackers/
24. Internet/
25. Internet-Based Intervention/
26. Multimedia/
27. Mobile Applications/
28. Online social networking/
29. Social Media/
30. Software/
31. Smartphone/
32. Technology/
33. Telehealth/
34. Telemedicine/
35. Telephone/
36. Television/
37. Text Messaging/
38. Video Games/
39. Virtual Reality/
40. Video Recording/
41. Wearable Sensors/
42. Webcasts/
43. App*.tx
44. Computer.tx
45. Digital.tx
46. DVD*.tx
47. eHealth.tx
48. e-Health.tx
49. Electronic.tx
50. EMail*.tx
51. Internet.tx
52. mHealth.tx
53. m-Health.tx
54. Mobile.tx
55. Multimedia.tx
56. Online.tx
57. Pedometer*.tx
58. Phone.tx
59. Sensor*.tx
60. Smartphone.tx
61. SMS.tx
62. “Social media”.tx
63. Social N media.tx
64. Social N network*.tx
65. Software.tx
66. Technolog*.tx
67. Telehealth.tx
68. Telemedicine.tx
69. Telephone.tx
70. Television.tx
71. Text.tx
72. Tracker*.tx
73. TV.tx
74. Video*.tx
75. Virtual.tx
76. (virtual or online) N (communit* or network*).tx
77. Wearable*.tx
78. Web*.tx
79. 18/79 OR
80. 7 AND 17 AND 79

PsycInfo Search

1. Gestational Diabetes/
2. Gestational diabetes.ti
3. Pregnancy induced diabetes.ti
4. Gestational diabetes mellitus.ti
5. GDM.ti
6. Pregnancy diabetes mellitus.ti
7. 1/6 OR
8. Preventive Health Behavior/
9. Health Education/
10. Preventative Health Services/
11. Health Risk Behaviour/
12. Health Knowledge/
13. Education*.ti
14. Intervention*.ti
15. Prevent* .ti
16. Program*.ti
17. Risk adj reduc*.ti
18. Risk.ti
19. 8/18 OR
20. Audiovisual communications media/
21. Computers/
22. Computer games/
23. Computer software/
24. Digital interventions/
25. Digital technology/
26. Digital video/
27. Electronic communication/
28. Electronic Health Services/
29. Electronic learning/
30. Internet/
31. Mobile applications/
32. Mobile devices/
33. Mobile learning/
34. Mobile health/
35. Mobile phones/
36. Multimedia/
37. Online community/
38. Online social networks/
39. Smartphones/
40. Social media/
41. Telemedicine/
42. Television/
43. Text messaging/
44. Videotapes/
45. Virtual Reality/
46. Wearable devices/
47. App*.mp
48. Computer.mp
49. Digital.mp
50. DVD*.mp
51. eHealth.mp
52. e-Health.mp
53. Electronic.mp
54. eMail*.mp
55. Internet.mp
56. mHealth.mp
57. m-Health.mp
58. Mobile.mp
59. Multimedia.mp
60. Online.mp
61. Pedometer*.mp
62. Phone.mp
63. Sensor*.mp
64. Smartphone.mp
65. SMS.mp
66. Social media.mp
67. Social adj media.mp
68. Social adj network*.mp
69. Software.mp
70. Technolog*.mp
71. Telehealth.mp
72. Telemedicine.mp
73. Telephone.mp
74. Television.mp
75. Text.mp
76. Tracker*.mp
77. TV.mp
78. Video*.mp
79. Virtual.mp
80. (virtual or online) adj/N (communit* or network*).mp
81. Wearable*.mp
82. Web*.mp
83. 20/83 OR
84. 7 AND 19 AND 83.

EMBASE search

1. Pregnancy diabetes mellitus/
2. Gestational diabetes.ti
3. Pregnancy induced diabetes.ti
4. Gestational diabetes mellitus.ti
5. GDM.ti
6. 1/5 OR
7. Health education/
8. Patient education/
9. Preventive medicine/
10. Risk reduction/
11. Education*.ti
12. Intervention.ti
13. Prevent*.ti
14. Program*.ti
15. Risk adj reduc*.ti
16. Risk.ti
17. 7/16 OR
18. Activity tracker/
19. Computer/
20. Mobile phone/
21. Digital Technology/
22. Distance learning/
23. DVD recorder/
24. E-mail/
25. Internet/
26. Mobile Applications/
27. Multimedia/
28. Online Social Network/
29. Smartphone/
30. Social Media/
31. Social Network/
32. Software/
33. Telemedicine/
34. Telephone/
35. Television/
36. Text Messaging/
37. Video Game/
38. Video Recording/
39. Virtual Reality/
40. Web browser/
41. Webcast/
42. App*.mp
43. Computer.mp
44. Digital.mp
45. DVD*.mp
46. eHealth.mp
47. e-Health.mp
48. Electronic.mp
49. eMail*.mp
50. Internet.mp
51. mHealth.mp
52. m-Health.mp
53. Mobile.mp
54. Multimedia.mp
55. Online.mp
56. Pedometer*.mp
57. Phone.mp
58. Sensor*.mp
59. Smartphone.mp
60. SMS.mp
61. Social media.mp
62. Social adj media.mp
63. Social adj network*.mp
64. Software.mp
65. Technolog*.mp
66. Telehealth.mp
67. Telemedicine.mp
68. Telephone.mp
69. Television.mp
70. Text.mp
71. Tracker*.mp
72. TV.mp
73. Video*.mp
74. Virtual.mp
75. (virtual or online) adj (communit* or network*).mp
76. Wearable*.mp
77. Web*.mp
78. 18/77 OR
79. 6 AND 17 AND 78

MIDRIS search

1. gestational diabetes.ti.
2. pregnancy induced diabetes.ti
3. gestational diabetes mellitus.ti
4. GDM.ti
5. 1/4 OR
6. education*.ti
7. intervention.ti
8. prevent*.ti
9. program*.ti
10. risk.ti
11. Risk adj reduc*.ti
12. 6/11 OR
13. app*.mp
14. computer.mp
15. digital.mp
16. DVD*.mp
17. ehealth.mp
18. e-health.mp
19. electronic.mp
20. email*.mp
21. internet.mp
22. mhealth.mp
23. m-health.mp
24. mobile.mp
25. multimedia.mp
26. online.mp
27. pedometer*.mp
28. phone.mp
29. sensor*.mp
30. smartphone.mp
31. SMS.mp
32. social media.mp
33. (Social adj media).mp
34. (Social adj network*).mp
35. software.mp
36. technolog*.mp
37. telehealth.mp
38. telemedicine.mp
39. telephone.mp
40. television.mp
41. text.mp
42. tracker*
43. TV.mp
44. video*.mp
45. virtual.mp
46. ((virtual or online) adj (communit* or network*)).mp
47. wearable*.mp
48. web*.mp
49. 13/48 OR
50. 5 AND 12 AND 49

| Concept 1 Medline | Concept 2 | 3 |  |
| --- | --- | --- | --- |
| Mesh Terms | Subject Headings |  |  |
| DiabetesMelllitus, Gestational/ |  |  |  |
|  |  |  |  |
| Free text |  |  |  |
|  |  |  |  |
|  |  |  |  |
|  |  |  |  |
